# 1-Aminocyclopropane-1-Carboxylate Deaminase-Producing Plant Growth-Promoting Rhizobacteria Improve Drought Stress Tolerance in Grapevine (*Vitis vinifera* L.)

**DOI:** 10.3389/fpls.2021.706990

**Published:** 2021-09-03

**Authors:** Bingbing Duan, Lin Li, Guoqiao Chen, Chenxing Su-Zhou, Yashan Li, Hasmik Merkeryan, Wei Liu, Xu Liu

**Affiliations:** ^1^College of Enology, Northwest A&F University, Yangling, China; ^2^School of Chemistry and Life Sciences, Chuxiong Normal University, Chuxiong, China; ^3^Horticulture Research Institute, Sichuan Academy of Agricultural Sciences, Chengdu, China; ^4^Ningxia Eastern Foot of Helan Mountain Wine Station, Northwest A&F University, Yinchuan, China

**Keywords:** 1-aminocyclopropane-1-carboxylate deaminase, drought stress, *Enterobacter* sp., plant growth-promoting rhizobacteria, *Pseudomonas* sp., *Vitis vinifera* L

## Abstract

Plant growth-promoting rhizobacteria (PGPRs) that produce 1-aminocyclopropane-1-carboxylate (ACC) deaminase are capable of reducing limits to plant growth due to water-deficient conditions. Here, seven PGPR strains that can produce ACC deaminase were successfully obtained from the rhizosphere soil of grapevine (*Vitis vinifera* L.) in arid regions of China. The strains belonged to three different genera: *Pseudomonas*, *Enterobacter*, and *Achromobacter*, according to their 16S rDNA sequencing analysis. A drought tolerance experiment revealed two PGPR strains (DR3 and DR6) with exceptionally high phosphate solubilization, nitrogen fixation, indoleacetic acid (IAA), and exopolysaccharides secretion potential. Both strains were selected for use in a pot experiment to evaluate their growth-promoting effects on grapevines under drought conditions. Each of these two PGPRs and their mixed inoculation into grapevines were expected to alleviate the comprehensive growth inhibition of grapevines caused by drought stress. The mixed inoculation was hypothesized to elicit the best growth-promoting effects. Inoculation with the PGPRs not only enhanced the root-adhering soil/root tissue ratios and soil aggregate stability, but it also increased the nitrogen and phosphorus levels in the soil and plant leaves. Further, inoculation with PGPRs significantly altered the plant height, biomass of shoot and root organs, relative water contents, and net photosynthetic rate of leaves, enabling grapevines to better cope with drought. Moreover, the contents of IAA, abscisic acid, and malondialdehyde in these grapevines under drought stress were significantly changed by PGPRs. They indirectly affected biochemical and physiological properties of grapevines to alleviate their drought stress. Taken together, these results demonstrate that the DR3 and DR6 PGPRs might be useful for effectively weakening the growth inhibition caused by drought in grapevines. The strains might also be applied as effective bioinoculants to maintain the quality of wine grapes.

## Introduction

With their continuous deterioration under global climate change, ecological systems are becoming seriously damaged. Drought, an environmental stress, is prevalent in arid regions, where it limits plant growth and threatens agricultural production (Vurukonda et al., 2016). As a root-borne stress, drought would normally lead to osmotic and oxidative stress, resulting in changed physiological, biochemical, and molecular properties of plants that jointly cause losses in crop production. In order to improve the drought tolerance of plants, various approaches have been explored, such as breeding drought-tolerant varieties and implementing water-saving irrigation (Evans and Sadler, 2008; Luo et al., 2019; Zhang et al., 2020). Yet, due to their advanced technological requirements and high labor and costs, certain approaches are not easy to apply in practice. Therefore, enhancing the drought tolerance of plants *via* targeted physical and biological methods is becoming a hot research topic in agricultural science.

Plant growth-promoting rhizobacteria (PGPRs) are bacterial groups obtained from rhizosphere soil. They can promote plant growth by means of biological control against soil-borne pathogens, biological nitrogen fixation, and root growth promotion (Pii et al., 2015). Therefore, they can cope with various environmental stresses and alleviate limitations to plant growth, effectively mitigating the loss of crop productivity. The growth-promoting effects of PGPRs on plants have been widely reported for many species (Mir et al., 2020; Zhang et al., 2020; Alemneh et al., 2021; Kalozoumis et al., 2021). Nevertheless, there are different genera of PGPRs as well as differences in the mechanisms by which they promote the growth of plants (Broadbent et al., 1977; Kaushal and Wani, 2016). PGPRs could directly and indirectly promote plant growth *via* nitrogen fixation, exopolysaccharides (EPS), and phytohormones (Vurukonda et al., 2016; Niu et al., 2018). Notably, 1-aminocyclopropane-1-carboxylate (ACC) deaminase-producing PGPRs, which are capable of hydrolyzing ACC to α-ketobutyrate and ammonia, could lower ethylene levels in plants and alleviate growth inhibition caused by excessive ethylene under environmental stressful conditions, drought in particular (Singh et al., 2015; Tiwari et al., 2018). Moreover, indoleacetic acid (IAA) is able to promote cell division and cell elongation, and thereby regulate root development and architecture (Spaepen et al., 2008; Naveed et al., 2015). The IAA content is reportedly increased after inoculation with PGPRs under environmental stress, and this alleviated exogenous stress incurred by the plants. In addition, EPS that attach to the surface of roots to form biofilms could protect these organs from drying out (Janczarek and Rachwał, 2013). Therefore, the mechanisms underpinning PGPRs’ alleviation of environmental stress and promotion of plant growth may be complicated, entailing a combination of many pathways (Cohen et al., 2015; Ortiz et al., 2015).

The wine grape (*Vitis vinifera* L.) plant is a particularly important perennial fruit vine growing in arid regions of China, where its production is a major economic activity. It is well known that the quality of wine depends on the grape berry’s chemical composition, which is the outcome of interactions between fruiting vines and their biotic and abiotic factors in their environment, such as local climate and soil physicochemical properties (Novello et al., 2017; Mazzei et al., 2019). Drought disturbances in arid regions have seriously impacted the quality of wine grapes and restricted the development of the grape industry (You et al., 2020). The rhizobacteria associated with grapevines in arid regions are prone to water shortages, and so they may have adapted to drought stress conditions; if so, they could help their host plants also adapt to drought stress. Some researchers did report on PGPRs isolated from grapevine rhizosphere soil that improved the quality of wine grapes (Aballay et al., 2011). Therefore, to cope with drought stress, it is imperative we try to isolate PGPRs for use in wine grape cultivation in these regions, to enhance both the quality of grapes produced and increase the incomes of local wine growers.

Accordingly, this study had three objectives: (1) to isolate PGPRs with ACC deaminase activity from the rhizosphere soil of wine grapevines; (2) to evaluate the effects of inoculations of selected PGPRs upon grapevines’ growth under imposed drought stress; and (3) to reveal the potential mechanisms of PGPRs’ alleviation of drought stress for grapevines by taking a comprehensive perspective. To the best of our knowledge, this is the first report to determine the effect and mechanism of ACC deaminase-producing PGPRs on grapevines under drought stress. We hoped to find effective PGPR strains for consideration as biological fertilizers for ameliorating drought stress incurred by grapevines in arid regions, to enable the sustainable development of the grape industry.

## Materials and Methods

### Isolation of Rhizobacteria With ACC Deaminase Activity

The rhizosphere soil used for the bacterial isolation was collected from 8-year-old vines of *V. vinifera* cv. Cabernet Sauvignon growing in the Xige winery on Helan Mountain, in Ningxia, China (38°3'44'N, 105°56'11'E), in 2018. Located in a BWk climate according to the Koppen-Geiger classification, this winery has a mean annual temperature of 10.8°C, maximum and minimum temperatures of 39°C and 21°C, respectively. The mean annual rainfall was 147mm, which mainly distributed from June to September. The annual reference evapotranspiration was 1208.5mm. The soil is loamy sand soil, classified as a Calcic Cambisol (FAO/UNESCO/ISRIC, 1988). The rhizosphere soils were obtained by gently shaking the plant roots; to isolate from them the bacteria with ACC deaminase activity, we followed Penrose and Glick’s (2003) method. Briefly, 1*g* rhizosphere soil was inoculated in a 50-ml sterile DF medium, which contained 3mm ACC as the sole nitrogen (N) source (Dworkin and Foster, 1958). Then, these media were incubated in a shaking incubator at 200rpm and 28°C for 24h. The ensuing isolates were kept for further use in Luria-Bertani slants (at 4°C).

### Bacterial Identification Using a 16S rDNA Sequencing Analysis

For each of the seven ACC deaminase-producing strains obtained, the 16S ribosomal DNA gene was amplified by PCR using the primers 27f (5'-AGAGTTTGATCCTGGCTCAG-3') and 1492r (5'-GGTTACCTTGTTACGACTT-3') at the genus level (Niu et al., 2018). The 16S rRNA sequences were determined with an ABI3730-XL DNA sequencer (Sangon Biotechnology Ltd., Shanghai, China). The detailed steps are described by Zhang et al. (2020). The obtained sequences were aligned and analyzed by the BLAST algorithm for comparison with published sequences in the National Center for Biotechnology Information nucleotide database,^1^ and submitted to GenBank. The phylogenetic tree based on16S rRNA sequencing analysis was constructed in MEGA v7.0, by using the neighbor-joining method applied to distance matrices.

### Analysis of Drought Tolerance and Plant Growth-Promoting Traits of Isolated Strains

The drought tolerance experiment of the isolated strains was fully described by Zhang et al. (2020). To evaluate the drought tolerance of isolates, different concentrations of polyethylene glycol 6000 were added to nutrient broth medium to create water potentials of different gradients (0, −0.05, −0.30, and −0.49MPa; Michel and Kaufmann, 1973). These media were inoculated with 1% bacterial strains isolated by ACC as the sole N sources and then incubated. Next, the growth-promoting traits of these strains were measured using the spectrophotometric method at 600nm. Phosphate solubilization was measured using method of the Mo-blue (Watanabe and Olsen, 1965; Chen et al., 2006). Döbereiner’s nitrogen-free medium was used to grow the bacterial strains to determine their putative N fixation ability (Day and Döbereiner, 1976; Cattelan et al., 1999). The IAA was detected using Salkowski’s reagent (Hassan et al., 2014). The EPS was measured using the phenol-sulfuric acid method (Khan et al., 2017). The ACC deaminase activity was detected by the method of Penrose and Glick (2003), whereby the amount of α-ketobutyrate degraded from ACC by isolated strains is monitored. According to the Bradford (1976) method, the protein content of toluenized cells was estimated.

### Pot Experiment Design

The pot experiment had a completely randomized design and was performed in a greenhouse from April to September in 2019 on the campus of Northwest A&F University, China (34°17'23'N, 108°4'14'E). Environmental conditions in the greenhouse during the drought stress were as follows: air temperature of 26–32°C, solar radiation of 300–600W/m^2^, reference evapotranspiration of 3–5mm/d, and vapor pressure deficit of 3.5–4.5Kpa. Based on the above results, *Pseudomonas corrugata* (DR3) and *Enterobacter soli* (DR6) were selected for this pot experiment. Separate inoculums of *P. corrugata* (DR3) and *E. soli* (DR6) were prepared by diluting the cultures to a final concentration of 10^8^CFU/ml. The soil for the pot experiment was also collected in the vineyard of Xige winery and classified as sandy loam, and heat-sterilized at 121°C for 3h (Barnawal et al., 2016b). Soil sterilization was applied to avoid interference of the native microbial communities and thus ameliorate colonizing efficiency of the inocula. Soil chemical properties for the pot experiment were as follows: pH of 8.03, organic matter (OM) content of 11.38g/kg, total nitrogen (TN) content of 0.51g/kg, total phosphorus (TP) content of 0.39g/kg, and an available P (AP) content of 4.35mg/kg.

For the bacterial inoculations, four treatments were implemented as follows: (1) *P. corrugate* alone (T1); (2) *E. soli* alone (T2); (3) a 1:1 volume mixture of *P. corrugata* and *E. soli* (T3); and (4) control without any bacteria (CK). For the drought treatment, plants were grown at two levels: at 75% field capacity [control, no drought stress (ND)] or 35% field capacity (drought stress, DS). Therefore, in this 2×4 factorial experiment, there was a total of 8 treatment combinations (4 bacterial inoculations×2 drought conditions), with 15 replicates of each treatment. To apply ACC deaminase-producing bacteria *P. corrugate*, *E. soli*, and their mixture, a syringe was used, through which 150ml of a given bacterial suspension was inoculated into the middle of a grapevine’s root system. The control grapevines received 150ml of ddH_2_O with no bacteria.

The Cabernet Sauvignon (*V. vinifera*) grapevines were obtained from the commercial vineyard of Xixia King Industry Co. Ltd. in Ningxia. These grapevines were cultivated with the hardwood cuttings using a conventional cutting propagation method in the last year. A 1-year-old own-rooted Cabernet Sauvignon grapevine was planted in each polyethylene pot (25cm diameter×30cm height) as one replicate in early April. The plants were subjected to drought stress in early August. Daily weighing of the pots was used to regulate the water content, and any lost water was replenished at 18:00 every day. The imposed drought conditions lasted for 25days, after which the soil and plant samples were collected for determination of the growth, physiological and biochemical characteristics of plants, and soil physicochemical properties to evaluate the plant growth-promoting effects of strains.

### Sample Collection

After grapevine growth for 25days under drought stress, plant photosynthetic characteristics were measured in early September. Then, soil and grapevines were all sampled for determination of various indices of soil and plants. Roots and shoots were separately collected by clipping grapevines at the soil surface and directly measured the plant height.

All roots were rinsed with distilled water for determination of root morphology. All the leaves were collected separately and divided into three subsamples: One sample was preserved in liquid nitrogen for antioxidant indicators, one sample was oven-dried at 75°C for leaf nutrients, and the third one that leaf at fifth or sixth nodes of shoots was for leaf relative water content (RWC). Soil samples were divided into two subsamples: One fresh soil sample was used to determine the potential activity of urease and alkaline phosphatase, and the other one sample was air-dried to measure the aggregate stability and nutrient contents.

### Dependent Variables Measured

The root-adhering soil/root tissue (RAS/RT) ratio was determined, as described by Sandhya et al. (2009). Distilled water was used to separate the RAS, after which the roots were dried at 105°C. Soil aggregate stability was then determined according to the amounts of water-stable aggregates (Chaudhary and Kar, 1972; Sandhya et al., 2009). Aggregate stability was assessed according to the amounts of water-stable aggregates. Soil available nitrogen (AN) was quantified using a continuous flow injection analyzer (Seal Auto Analyzer 3-AA3; Zhang et al., 2016). Soil AP was determined by the molybdenum antimony colorimetric method, following the methodology used by Page et al. (1982). The potential activity of urease and alkaline phosphatase was, respectively, assayed using the phenol sodium standard colorimetric and disodium phenyl phosphate standard colorimetric method (Saiya-Cork et al., 2002).

Plant growth characteristics were analyzed by determining the height and stem diameters, biomass of shoot and root parts, root parameters, leaf nutrients, and RWC. Plant height and diameter were measured on a metric scale. To obtain the shoot and root biomass, they were weighed after being dried in a forced hot-air oven at 70°C for 2days (Ryle et al., 1981). Root morphology was examined by scanning the roots on a flatbed scanner (EPSON, Perfection V-750), for which total root length, root surface area, and volume were obtained for each plant by analyzing the root images in WinRHIZO Arabidopsis 2017a software (WinRHIZO, RRID:SCR_017120; Oddiraju et al., 1996; Himmelbauer et al., 2004).

For leaf nutrients, nitrogen and phosphorus contents were quantified using the method described by Guo et al. (2005) and Chandra et al. (2019). Leaf sample (0.20g) was weighed and extracted with H_2_SO_4_-HClO_4_. The content of TN was determined at 645nm by indophenol blue colorimetry, and the content of TP was measured by molybdenum antimony colorimetry. RWC of leaves was determined using the methodology of Meher et al. (2018) and calculated according to Sharp et al. (1990). Briefly, a total of 10 leaves per treatment were weighed and immersed in distilled water at 4°C for 12h. The leaves were then dried the water on the surface and re-weighed as saturated fresh weight. The immersed leaves were placed in an aluminum box and baked at 105°C for 15min, and then dried at 75°C to constant weight. When cool to room temperature, the leaves were weighed again to obtain dry weight.

The chlorophyll in leaves was extracted in 10ml of 80% (v/v) acetone and then measured at the wavelengths of 663nm and 645nm by spectrometry, respectively (Calvo-Polanco et al., 2016). Plant photosynthetic characteristics of 10 leaves at sixth or seventh nodes of shoots for each treatment were also evaluated at 9:00–11:00am on the day after drought stress was finished. Their photosynthetic rates (Pn) and stomatal conductance (Gs) were quantified with a portable photosynthesis system (LI-COR-6400, Lincoln, NE, United States). The flow rate of air was 750μmol/min. The light source was red and blue LEDs with 1800μmol/(m^2^s) of light intensity. The water use efficiency (WUE) was calculated according to the photosynthetic parameters.

The phydetek-IAA and phydetek-abscisic acid (ABA) immunoassay kits (Agdia, Elkhart, IN, United States) were, respectively, used to determine the IAA and ABA contents of leaves. The contents of these two phytohormones were determined based on the manufacturer’s protocol.

The grapevines were also used for an assessment of key antioxidant indicators, namely, their malondialdehyde (MDA), superoxide dismutase (SOD), peroxidase (POD), and catalase (CAT) levels. MDA content was measured following Heath and Packer (1968). Briefly, leaves tissue (0.50g) was added into 2ml of 0.1% trichloroacetic acid (v/v) and ground into homogenate, and then was extracted with 5ml of 0.5% thiobarbituric acid (v/v) solution. The extract solution was shaken, boiled at boiling water bath for 10min. After cooling to room temperature, the resulting extraction was centrifuged, and then, the suspension was determined at the 532nm and 600nm on a spectrophotometer, respectively.

The SOD, POD, and CAT enzymes were extracted using the method described by Li et al. (2017). Briefly, leaves tissue (0.50g) was homogenized with precooled 1.5ml of Tris-HCl buffer (pH 7.5), containing 0.1% mercaptoethanol (v/v), and 5% sucrose (w/v). The homogenate was then centrifuged, and the supernatants of crude enzymes were used to determine the activity of SOD, POD, and CAT. SOD activity was determined by Beyer and Fridovich (1987). One unit of SOD activity was defined as the amount of enzyme required to inhibit the reduction rate of nitroblue tetrazolium by 50% at 25°C. POD activity was measured according to the previous method (Zhou and Leul, 1998). According to Beer and Sizer (1952), CAT activity was determined by monitoring the disappearance of H_2_O_2_ at 240nm.

### Statistical Analysis

One-way ANOVA was used to analyze the differences of the data with SPSS 19.0. Duncan’s multiple range tests compared the means of these variables in a pairwise manner at a significant level of *P*<0.05. The graphs were drawn in OriginPro 9.0.

## Results

### Isolation and Identification of Bacterial Strains With ACC Deaminase Activity

**Figure 1:**
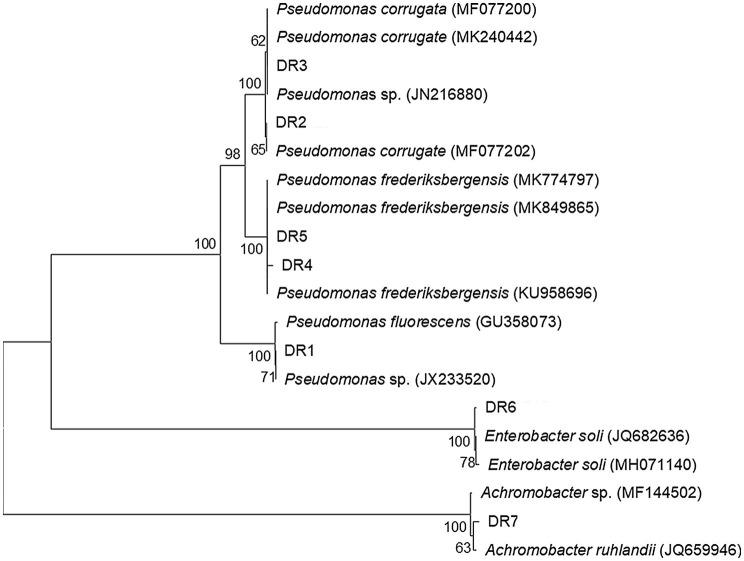
Phylogenetic tree of seven ACC deaminase-producing strains isolated from the rhizosphere of grapevines. Distance and clustering analyses were performed, using the neighbor-joining method, in MEGA v7.0. Bootstrap values (*n*=1,000) are given as percentages at the branching points.

Seven ACC deaminase-producing bacterial strains were obtained from the rhizosphere soil of grapevine. These isolated bacterial strains differed in their ability to produce ACC deaminase (Table 1). The ACC deaminase activity of these two stains, isolate DR3 and DR6, was higher than the left five stains. Notably, the ACC deaminase activity of isolate DR3 was 60.11μmol α-KB/(mgPrh), which was about 50% higher than the 41.18μmol α-KB/(mgPrh) produced by isolate DR6. Next, these seven ACC deaminase-producing bacterial strains were identified by the 16S rDNA sequencing analysis; their similarity with the closest known type strains was 99–100% (Table 1). The phylogenetic analysis revealed these strains belonged to three different genera: *Pseudomonas* (five isolates), *Enterobacter* (one isolate), and *Achromobacter* (one isolate; Figure 1).

**Table 1:** 1-aminocyclopropane-1-carboxylate (ACC) deaminase activity of the isolated bacteria associated with grapevine rhizosphere soil.

Isolate no.	Genera	Nearest-type strain	Accession no.	Similarity (%) of the 16S rRNA gene	ACC deaminase activity [μmolα-KB/(mgPrh)]
DR1	*Pseudomonas fluorescens*	GU358073	MK774791	99.72%	15.69±0.31g
DR2	*Pseudomonas corrugata*	MF077202	MK346043	99.86%	22.87±0.60e
DR3	*P. corrugata*	MK240442	MK411217	99.93%	60.11±0.94a
DR4	*Pseudomonas frederiksbergensis*	MK849865	MK611658	99.72%	26.21±0.66d
DR5	*Pseudomonas frederiksbergensis*	KU958696	MK611660	99.86%	34.25±0.64c
DR6	*Enterobacter soli*	JQ682636	MK611659	99.51%	41.18±0.93b
DR7	*Achromobacter xylosoxidans*	JQ659946	MK611665	99.72%	19.39±0.45f

Values are the mean±*SE* (*n*=3). Different lowercase letters indicate significant differences according to Duncan’s multiple range test at the *P*<0.05 level.

### Drought Tolerance, Plant Growth-Promoting Properties, and EPS Production of Isolates

Although all seven isolates had nitrogen fixing, phosphate solubilization, and IAA secretion abilities, the three isolates DR1, DR3, and DR6 were able to survive under drought conditions of −0.30MPa water potential (Table 2). Furthermore, all strains except DR7 had the ability to secrete EPS, while DR3 and DR6 were distinguished by higher phosphate solubilization, nitrogen fixation, and EPS secretion potential (Table 2). For strain DR3, its phosphate dissolving capacity and EPS production were, respectively, 64.07μg/ml and 9.43mg/mg protein, with slight lower corresponding values for strain DR6, at 58.97μg/ml and 7.38mg/mg protein. Further, DR3 and DR6 harbored the greatest IAA production ability (at 10.29 and 11.14μg/ml, respectively). Hence, the DR3 and DR6 strains were used in the pot experiment.

**Table 2:** Plant growth-promoting characteristics of the isolates from grapevine rhizosphere soil.

Strain	Drought tolerance (OD_600_ at −0.30MPa)	Indoleacetic acid (IAA) production (μg/ml)	Nitrogen fixation	Phosphorus-solubilizing circle (mm)	Phosphate dissolving (μg/ml)	Exopolysaccharides production (mg/mg protein)
DR1	0.25	4.46±0.27f	+	6.51±0.29d	49.37±0.39e	4.10
DR2	–	8.33±0.44cd	+	7.62±0.30d	52.25±0.64d	4.44
DR3	1.05	10.29±0.21b	+	14.17±0.08a	64.07±0.79a	9.43
DR4	–	9.08±0.20c	+	9.91±0.24c	57.06±0.48c	4.16
DR5	–	8.07±0.13d	+	7.36±0.13d	51.89±0.33d	3.52
DR6	0.84	11.14±0.11a	+	11.65±0.91b	58.97±0.17b	7.38
DR7	–	5.44±0.41e	+	7.60±0.11d	51.84±0.28d	–

Values are the mean±*SE* (*n*=3). Different lowercase letters indicate significant differences according to Duncan’s multiple range test at the *P*<0.05 level.

### RAS/RT Ratio, Soil Aggregate Stability, Soil Nutrient Contents, and Related Enzyme Activity in Pot Experiment

Drought stress significantly increased both the RAS/RT ratio and soil aggregate stability (Table 3). Moreover, PGPRs also positively influenced the RAS/RT ratio and soil aggregate stability. Under drought, the RAS/RT ratios of treatments with inoculations of T1, T2, and T3 were, respectively, increased by 25.52, 29.35, and 56.47% over the control group; their corresponding soil aggregate stabilities were increased by 19.65, 22.66, and 39.68%. The RAS/RT ratios and soil aggregate stability were significantly higher in the mixed inoculation treatment (T3) than with the inoculation of either bacterial strain alone (*p*<0.05).

**Table 3:** Effects of inoculation with ACC deaminase-producing plant growth-promoting rhizobacteria (PGPRs) on root-adhering soil/root tissue (RAS/RT) ratio, soil aggregate stability, available N, available P and enzyme activities under non-drought (ND) and drought stress (DS) conditions.

Drought stress	Treatment	RAS/RT	Aggregate stability (%)	Available N (mg/kg)	Available P (mg/kg)	Urease (mg/g)	Alkaline phosphatase (mg/kg)
ND	CK	23.52±0.70d	34.75±0.84d	30.96±0.84c	4.23±0.11d	1.34±0.07c	14.75±0.84d
	T1	30.27±1.13c	42.56±2.04c	38.71±1.61b	5.47±0.28b	1.82±0.18b	22.56±1.61c
	T2	31.17±2.59c	42.95±0.87c	39.04±0.87b	5.67±0.08b	1.91±0.04b	25.95±0.87b
	T3	32.02±1.26c	49.66±2.64b	47.90±0.91a	6.52±0.13a	2.67±0.04a	37.66±0.91a
DS	CK	33.15±0.97c	43.20±1.17c	20.47±0.66e	2.60±0.06f	0.88±0.08d	9.20±0.66e
	T1	41.61±1.87b	51.69±1.90b	26.16±0.94d	3.13±0.05e	1.35±0.19c	14.19±0.94d
	T2	42.88±1.70b	52.99±2.19b	30.97±1.04c	3.31±0.06e	1.40±0.04c	16.99±1.04d
	T3	51.87±2.73a	60.34±1.45a	39.61±0.88b	4.72±0.08c	1.97±0.12b	24.34±0.88bc

Values are the mean±*SE* (*n*=3). Different lowercase letters indicate significant differences according to Duncan’s multiple range test at the *P*<0.05 level.

The soil available N and available P contents were decreased significantly by drought stress, but this was alleviated after inoculation with PGPRs (Table 3). Compared with the control (CK) under drought stress conditions, the AN and AP contents under inoculation with PGPRs were increased considerably, by 27.80–93.50% and 20.38–81.84%, respectively. The soil AN content (39.61mg/kg) and AP content (4.72mg/kg) under the T3 inoculation exceeded those in the other treatments (*p*<0.05), under both non-drought and drought stress conditions. Similar patterns characterized the urease and alkaline phosphatase responses (Table 3). Under drought, the urease activity of the T1, T2, and T3 inoculation treatment increased by 53.41, 59.09, and 123.86%, respectively; the corresponding increases for alkaline phosphatase activity were 54.24, 84.67, and 164.57%. Thus, inoculating the rhizosphere with PGPRs can increase the local availability of soil nutrients and reduce the impact of a drought condition on grapevines.

### Growth and Photosynthetic Characteristics Responses of Grapevines

Plant height, shoot, root, and leaf growth parameters were all decreased by drought stress (Table 4). However, these negative effects of drought stress were ameliorated after inoculation with different strains, especially the mixed inoculation (T3). Plant height at mixed inoculation (T3) under no drought stress and drought stress were 73.84cm and 69.46cm, respectively. Compared with the control, the inoculation of the PGPR strains under drought stress increased plant height by 12.30–32.68%; moreover, the dry weight of shoot and root components was, respectively, increased by 19.84–36.64%, and 28.03–64.74%. In addition, the strains significantly improved the diameter of grapevines’ shoot, as well as the length, surface area, volume, and activity of their roots. In particular, the T3 treatment increased the root activity by 115.12%, directly affecting plant growth and nutrient contents.

**Table 4:** Effects of inoculation with ACC deaminase-producing PGPRs on the growth parameters and nutrient uptake of grapevines under non-drought (ND) and drought stress (DS) conditions.

Drought stress	Treatments	Plant height (cm)	Shoot		Root					Leaf		
			DW^a^ (g)	Diameter (mm)	DW (g)	Length (cm)	Surface area (cm^2^)	Volume (cm^3^)	Activity (μg/g·h)	TN^b^ (g/kg)	TP^c^ (g/kg)	RWC^d^ (%)
ND	CK	59.33±1.17de	5.79±0.04c	4.45±0.21c	4.99±0.13cd	3,192±83c	706±9c	10.42±0.43d	63.81±1.32d	13.69±0.07f	2.43±0.15d	80.29±0.76c
	T1	66.25±1.51bc	6.84±0.50b	5.43±0.14b	5.84±0.17b	3,302±92bc	767±21b	15.62±0.72b	75.96±4.74c	19.71±0.18d	3.54±0.16c	87.10±0.65b
	T2	68.29±1.21b	7.02±0.38b	5.56±0.16b	5.99±0.29b	3,465±57b	789±8b	17.25±1.73b	87.21±1.03b	22.04±0.04c	4.93±0.46b	87.78±0.80b
	T3	73.84±1.81a	8.04±0.21a	6.18±0.23a	7.70±0.22a	3,843±54a	863±14a	20.98±0.69a	103.18±1.52a	29.92±0.04a	6.40±0.34a	92.39±0.75a
DS	CK	52.35±1.15f	4.94±0.09d	3.67±0.12d	3.46±0.07f	2,277±85e	508±5f	6.76±0.47e	29.97±0.62f	11.01±0.08g	1.58±0.26e	62.48±0.81e
	T1	58.79±1.47e	6.68±0.05b	4.52±0.18c	4.43±0.52d	2,588±56d	566±10e	10.38±0.44d	51.03±3.85e	16.60±0.19e	2.67±0.28d	71.96±0.56d
	T2	63.40±1.21cd	5.92±0.15c	4.68±0.17c	4.79±0.24d	2,715±51d	636±14d	10.57±0.33d	57.27±3.91de	19.65±0.04d	3.96±0.12c	74.50±1.04d
	T3	69.46±1.98b	6.75±0.08b	5.39±0.12b	5.70±0.17bc	3,199±104c	705±8c	13.07±0.33c	64.47±1.22d	23.23±0.12b	5.06±0.30b	81.89±1.24c

a DW: dry weight.

b TN: total nitrogen.

c TP: total phosphorus.

d RWC: relative water content.

Values are the mean±*SE* (*n*=3). Different lowercase letters indicate significant differences according to Duncan’s multiple range test at the *P*<0.05 level.

The growth and photosynthetic characteristics of grapevines were markedly decreased while under drought stress (Tables 4 and 5), whereas inoculation of the strains was able to effectively counter this impact. Leaf total nitrogen, total phosphorus, RWC, chlorophyll contents, Pn, Gs, and WUE were all increased at treatments with inoculations of the strains by 50.77–110.99%, 68.99–220.25%, 15.17–31.07%, 31.63–61.22%, 30.04–61.31%, 52.89–198.10%, and 41.67–103.13%, respectively, when compared with the control under drought stress. Among the three treatments, the mixed inoculation (T3) best improved the growth and photosynthetic characteristics of grapevines.

**Table 5:** Effects of inoculation with ACC deaminase-producing PGPRs on the chlorophyll contents, Pn, Gs, and water use efficiency (WUE) of grapevine leaves under non-drought (ND) and drought stress (DS) conditions.

Drought stress	Treatments	Chlorophyll contents (mg/g)	Pn^a^ (μmol/m^2^s)	Gs^b^ (mmol/m^2^s)	WUE^c^ (μmol/mmol)
ND	CK	2.62±0.05c	8.01±0.55cd	80.73±3.58d	1.40±0.12d
	T1	3.05±0.03b	10.11±0.47b	93.46±3.64c	2.52±0.16b
	T2	3.09±0.05b	10.27±0.40b	112.10±4.34b	2.57±0.11b
	T3	3.43±0.08a	11.88±0.38a	144.78±5.68a	3.04±0.16a
DS	CK	1.96±0.01d	5.66±0.49e	26.85±2.45g	0.96±0.08e
	T1	2.58±0.07c	7.36±0.49d	41.05±2.93f	1.46±0.11d
	T2	2.71±0.10c	7.02±0.31d	66.51±3.78e	1.36±0.11d
	T3	3.16±0.15b	9.13±0.52bc	80.04±3.61d	1.95±0.12c

a Pn: photosynthetic rates.

b Gs: stomatal conductance.

c WUE: water use efficiency.

Values are the mean±*SE* (*n*=3). Different lowercase letters indicate significant differences according to Duncan’s multiple range test at the *P*<0.05 level.

### IAA and ABA Contents

**Figure 2:**
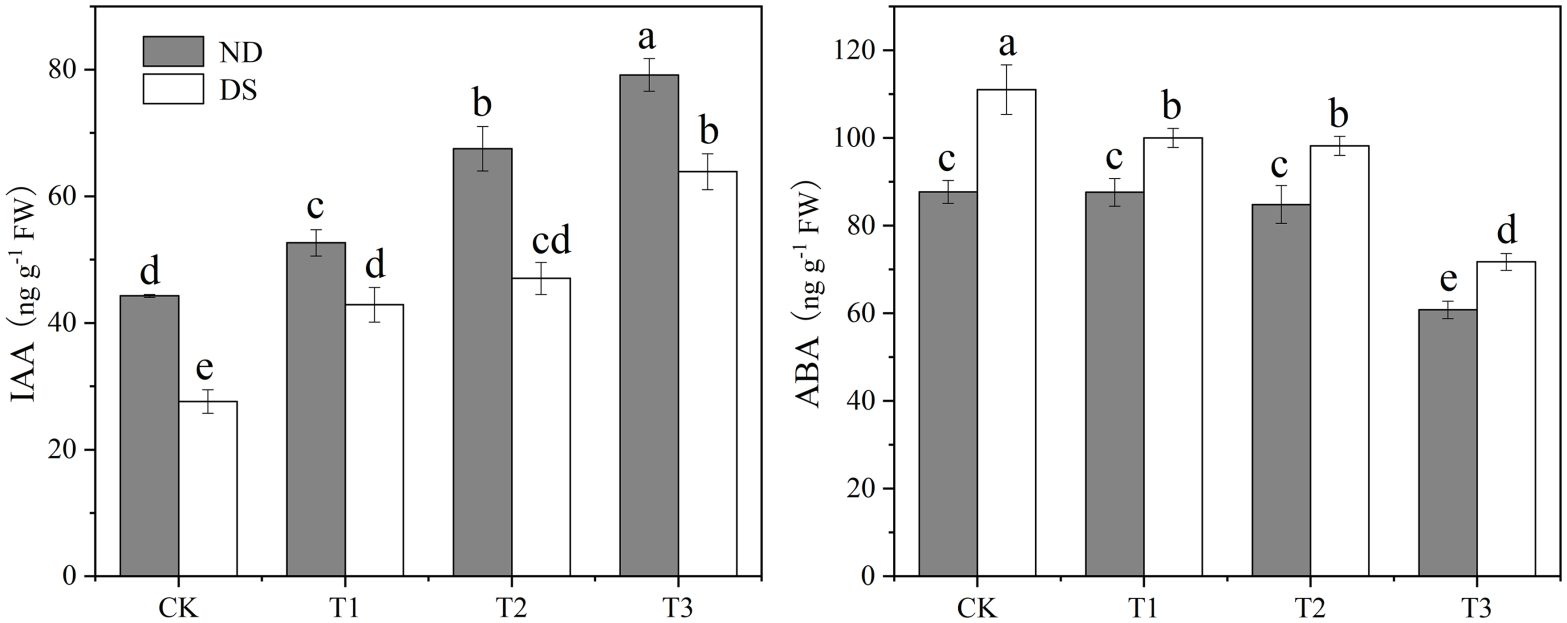
Effects of inoculation with ACC deaminase-producing PGPRs on IAA and ABA contents of grapevine leaves under non-drought (ND) and drought stress (DS) conditions. Bars represent the mean±SE (n = 3).

Drought stress and the bacterial strains’ inoculation significantly influenced the phytohormones, IAA, and ABA of grapevines (Figure 2). Their IAA content was significantly decreased by drought stress, but it was significantly 18.9–78.8% and 55.2–131.4% higher in the PGPR inoculation treatments than the controls without and with drought stress, respectively. Among the inoculation treatments under both non-drought and drought stress conditions, the IAA content of grapevines inoculated with T3—the mixed inoculation of *P. corrugate* and *E. soli*—was always the highest, while their ABA contents were the lowest. However, the ABA content of grapevines was significantly increased by drought stress. Compared with the control under drought stress, ABA contents of inoculation treatments T1 (*P. corrugata*), T2 (*E. soli*), and T3 (*P. corrugate* and *E. soli*) were reduced by 9.94, 11.54, and 35.42%, respectively.

### MDA Content and Antioxidant Enzymes Activity

**Figure 3:**
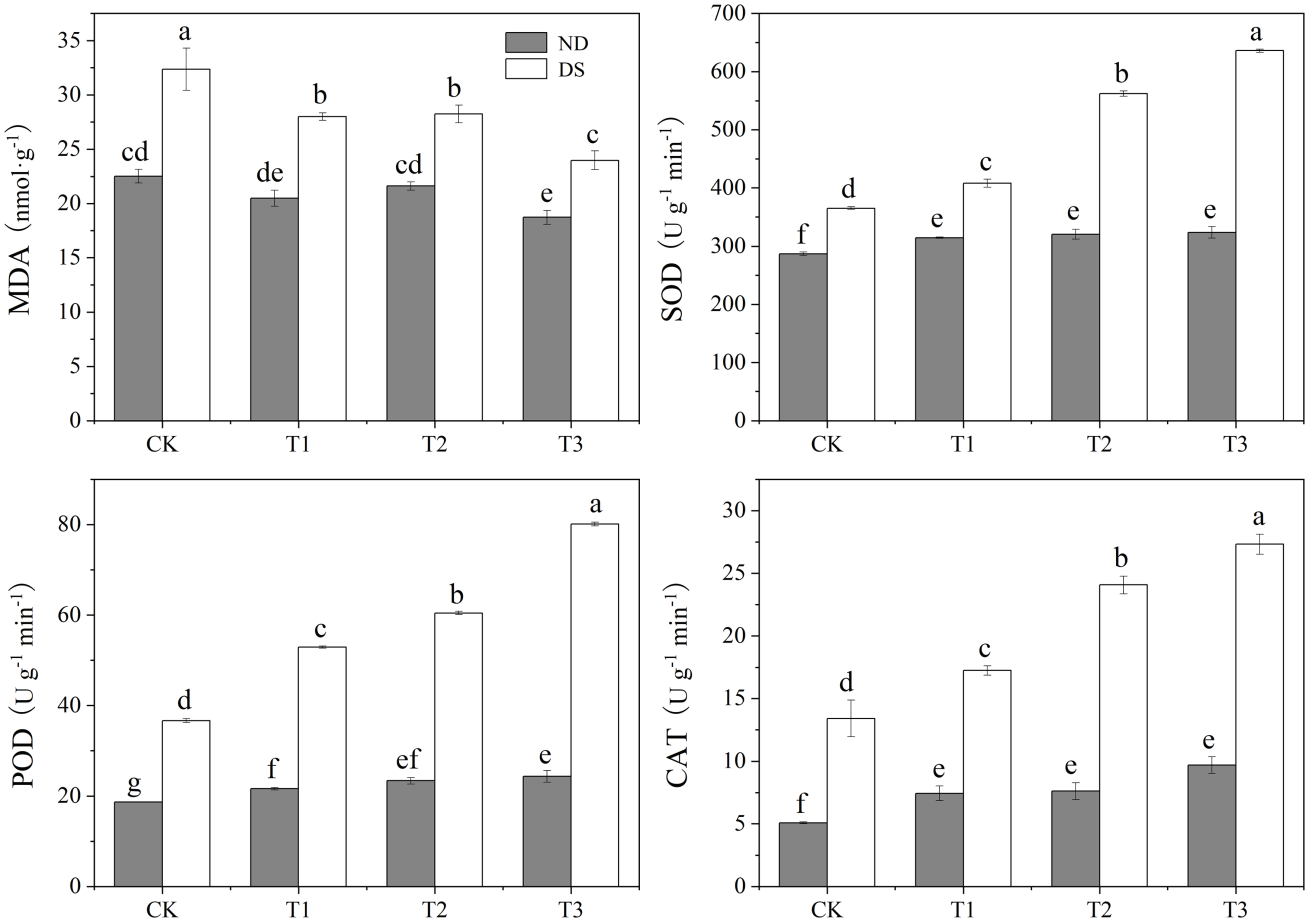
Effects of inoculation with ACC deaminase-producing PGPRs on the malondialdehyde (MDA) content and antioxidant enzymes activity of grapevine leaves under non-drought (ND) and drought stress (DS) conditions. Bars represent the mean±SE (n = 3).

Drought stress caused a significant increment in MDA content and augmentation in the activity of antioxidant enzymes (SOD, POD, and CAT; Figure 3). Compared with the non-inoculated grapevines, the MDA content decreased by 12.7–25.9%, and the SOD, POD, and CAT activities improved by 11.8–74.1%, 44.2–118.4%, and 28.5–103.6%, respectively. Under drought, the MDA content was significantly decreased by the PGPR inoculations, reaching its lowest value, 23.98nmol/g, under T3. Conversely, antioxidant enzymes (SOD, POD, and CAT) activities were significantly increased after the inoculation with PGPRs, attaining their highest values under T3. These results showed that PGPR inoculations could reduce the accumulation of MDA in leaves of grapevines under drought conditions, thereby lessening the severity of incurred membrane damage and improving their drought resistance.

### Soil and Plant Growth Properties Associated With the Phytohormones and MDA

**Figure 4:**
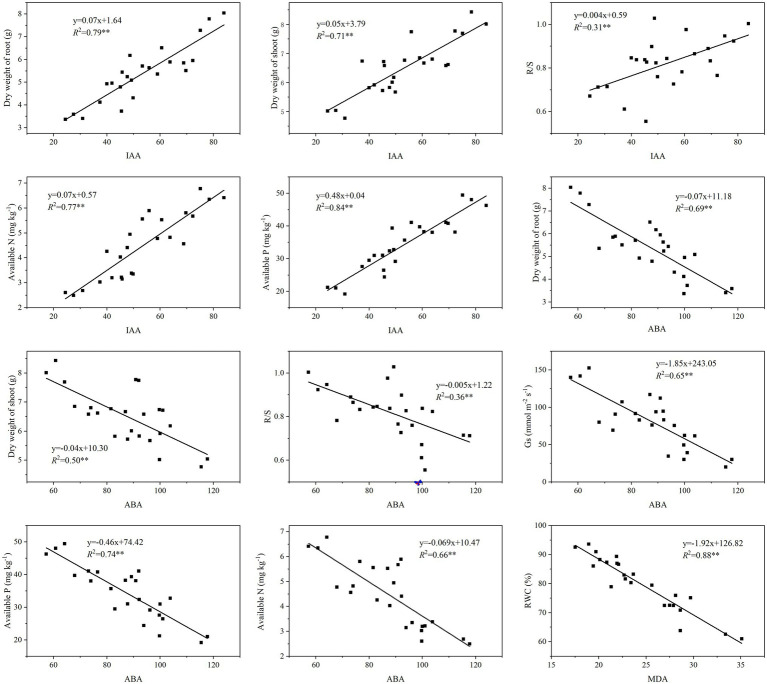
Linear regressions showing effects on the physiological and biochemical properties of grapevines from the phytohormones and MDA that were altered by inoculation with ACC deaminase-producing PGPRs.

The phytohormones IAA and ABA, and MDA, each had significantly positive correlations with the grapevines’ root and shoot properties and photosynthetic characteristics, and the soil properties (Table 6). Yet, only SOD, POD, and CAT were positively correlated with soil properties. Specifically, IAA was positively correlated with the dry weight of root as well as the dry weight of shoot and the R/S ratio (Figure 4). In addition, IAA content was also positively correlated with the soil AN and AP contents. By contrast, ABA was negatively correlated with the dry weight of root and shoot, the R/S ratio as well as Gs, and also AN and AP. Lastly, MDA was negatively correlated with RWC.

**Table 6:** Mantel test results for the correlations between the grapevines’ phytohormones, MDA, and antioxidant enzymes and their root, shoot, photosynthesis, and soil properties.

Parameters	Root properties		Shoot properties		Photosynthetic properties		Soil properties	
	*r*	*P*	*r*	*P*	*r*	*P*	*r*	*P*
IAA	**0.66**	**0.00**	**0.80**	**0.00**	**0.62**	**0.00**	**0.57**	**0.00**
ABA	**0.69**	**0.00**	**0.68**	**0.00**	**0.55**	**0.00**	**0.53**	**0.00**
MDA	**0.89**	**0.00**	**0.75**	**0.00**	**0.76**	**0.00**	**0.50**	**0.00**
SOD	−0.05	0.65	−0.08	0.77	−0.08	0.79	**0.34**	**0.00**
POD	0.09	0.15	0.01	0.39	0.03	0.31	**0.46**	**0.00**
CAT	0.04	0.28	−0.03	0.60	−0.02	0.52	**0.41**	**0.00**

Bold numbers indicate significant correlations (*P*<0.05).

## Discussion

The PGPRs with ACC deaminase activity could reduce ethylene’s inhibition of plant growth and induce plant stress resistance *via* phytohormone signaling pathways, thus positively impacting plant growth and alleviating abiotic stresses, like drought. Unsurprisingly, such PGPRs are sought/isolated and widely studied for various crops to help them cope with adverse growing conditions. In this study, a total of seven ACC deaminase-possessing strains from grapevine rhizosphere soil in the arid region were isolated. Their ACC deaminase activities varied almost 4-fold, from 15.69 to 60.11μmol α-KB/(mgPrh). The levels of ACC deaminase activity measured in our study are generally higher than those of strains isolated from rhizosphere soil of other crops, such as 1.89–39.40μmol α-KB/(mgPrh) in foxtail millet (Niu et al., 2018) and 1.82–41.58μmol α-KB/(mgPrh) in wheat (Ansari et al., 2021). These differences may be due to the different species studied, as well as the degree of stress incurred by the host plants (Niu et al., 2018), given that an environmental stress likely induces the strain to develop tolerance to that stress (Hoffmann and Hercus, 2000). More than 20 genera of rhizosphere bacteria are now known to harbor potential plant disease prevention and growth promotion benefits, such as *Pseudomonas*, *Bacillus* (Broadbent et al., 1977), *Agrobacterium*, and *Eriwinia.* Among these genera, *Pseudomonas* is the dominant genus, accounting for 60–93% of the PGPRs identified to date. Further, the genera of isolated strains mainly depend on the species identity of their host plants (Gontia-Mishra et al., 2017; Niu et al., 2018); for example, *Pseudomonas* and *Arthrobacter* were isolated from rhizosphere soil of millet, and likewise *Acinetobacter* in addition to *Bacillus* from that of wheat (Timmusk et al., 2014; Gontia-Mishra et al., 2017). Among the seven isolated strains in our study, at a frequency of 74.1%, *Pseudomonas* clearly was the dominant class in the rhizosphere soil of grapevine. Further, of the seven strains, two strains (*P. corrugata* DR3 and *E. soli* DR6) not only could survive under the −0.3MPa condition but also featured the greatest ACC deaminase activity (DR3: 60.11μmol α-KB/(mgPrh); DR6: 41.18μmol α-KB/(mgPrh)). This key trait could, to a considerable extent, relieve the ethylene stress to plants induced by drought, along with positively affecting their growth under drought conditions. Therefore, these two strains were used in the pot experiment to test the effects of PGPR on grapevines and discern their mechanisms.

In this study, drought decreased the plant height, the dry matter and diameter of shoots, the root length and volume, and leaf nutrient and photosynthetic characteristics, thus impairing the growth of grapevines. These results are supported by other findings that drought can alter physiological, biochemical, and molecular processes in plants, leading to productivity losses (Kaushal and Wani, 2016). Some studies reported that a PGPR inoculation could modulate key morphological and biochemical processes to mitigate the drought stress incurred by crop plants or herbs, such as maize (Jochum et al., 2019), wheat (Yaseen et al., 2019), and *Chlorophytum* (Barnawal et al., 2016a). Accordingly, in the present study, the growth traits and physiological properties of grapevines were measured under non-drought and drought stress conditions. This demonstrated that single-PGPR inoculations of *P. corrugata* or *E. soli* promoted the plant growth. Importantly, applying the mixture inoculation of both PGPRs had stronger promotion effects than applying either alone. Specifically, plant height, shoot dry matter and diameter, root length and volume, and leaf nutrients were all significantly higher for the mixture of PGPRs than either single-strain inoculation or none at all (control) under drought condition. These results are consistent with other findings (Jochum et al., 2019; Zhang et al., 2020). For example, inoculation with PGPRs improved the growth of spring wheat plants (Baris et al., 2014), whose dry matter content and growth were higher when treated with the mixed than single inoculations (Şahin et al., 2004; Baris et al., 2014). Therefore, two PGPRs, *P. corrugata* and *E. soli*, are capable of effectively enhancing the drought tolerance of grapevine and ameliorating the negative effects to it caused by drought.

**Figure 5:**
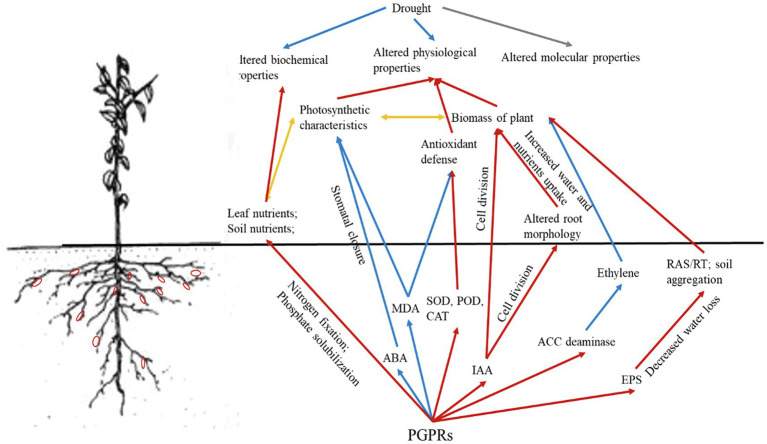
Conceptual mechanistic framework of the coordinated regulation of drought stress response and PGPRs with ACC deaminase activity in grapevines under drought. Red arrows represent positive impacts; blue arrows represent negative impacts; yellow arrows represent interactions; and the gray arrow was not relevant to the present study.

Although both PGPRs *P. corrugata* and *E. soli* were isolated, selected, and inoculated to improve drought tolerance and growth of grapevine plants, their mechanisms may nonetheless be complicated and likely involve a complex combination of many pathways (Cohen et al., 2015; Ortiz et al., 2015). For example, PGPRs are known to benefit plant growth under drought conditions in multiple ways (Figure 5), because of their role in various processes: absorption of nutrients and water, root proliferation, aggregate stability, EPS production, and regulating phytohormone secretion (Sandhya et al., 2009; Khan et al., 2017). Nitrogen (N) participates in chlorophyll synthesis, so higher leaf concentrations of N could improve the photosynthetic rate of plants (Chen et al., 2005). Phosphorus (P) could participate in energy synthesis and promote plant growth, which are both determined by the relative supply of N and P in soil (Longstreth and Nobel, 1980; Mattos et al., 2003). Here, adding PGPRs augmented the available N and P contents, along with N, P-related enzyme activities and urease and alkaline phosphatase activities, to promote the 1-year-old own-rooted grapevine growth; however, the mechanisms responsible are different. We know that PGPRs capable of nitrogen fixation and P-solubilization can promote plants’ absorption of specific nutrients and increase their utilization rate of nutrients (Islam et al., 2014; Wang et al., 2020). In this way, PGPRs that promote nitrogen fixation will enhance the uptake of N by plants, thereby increasing the N content and photosynthetic rate of leaves. These positive effects on plants would feedback belowground, leading to more plant root exudates being formed and released into the rhizosphere, including ACC. Meanwhile, those PGPRs with ACC deaminase will decompose ACC as an N resource. This could drive a concentration gradient of ACC between the internal and external plant roots, and continuously promote ACC exudation from roots, which would also mitigate the ethylene stress induced by drought.

Because P typically has only one source—mainly from the weathering of bedrock material—the relatively low P content of soil would restrict plant growth (Del Campillo et al., 1999; Ezawa et al., 2002; Vassilev and Vassileva, 2003). Regarding the increased available P contents in soil in this study, it is likely driven by the P-solubilization of PGPRs, this increasing the production of P-related enzymes by PGPRs, which stimulates the availability of P in soil, as suggested by our Table 5 results. These findings are supported by several studies (Vacheron et al., 2013; Bargaz et al., 2018; Enebe and Babalola, 2018). Moreover, some work has shown that *Pseudomonas* and *Escherichia coli* used in present study function well as phosphate solubilizers (Park et al., 2008). Compared with the single strain, the inoculation of mixed strains had a more pronounced effect on soil fertility. This further proved that applying PGPRs could improve the nutrient contents of soil and promote the absorption and utilization of soil nutrients by plants. Therefore, inoculation with PGPRs could be used as good biofertilizers to regulate the soil nutrient elements and improve drought resistance of grapevines under drought conditions.

Drought is a root-borne stress, because the corresponding root metabolism that occurs under drought mainly will influence the photosynthesis process. Therefore, the alternations to root system architecture under drought conditions are a response best understood as a stress defensive mechanism. The EPS, a high molecular weight type of carbohydrate, could help bacteria attach themselves to the plants root surface for the formation of biofilm which protect the roots from drying (Janczarek and Rachwał, 2013) and improves their drought tolerance. In addition, research has shown that EPS-producing PGPR could improve water uptake and soil nutrients absorption by improving the RAS/RT ratio and macro-aggregate stability under drought conditions (Janczarek et al., 2015). Our results showed that single and mixed inoculations of EPS-producing DR3 and DR6 into the rhizosphere did not only significantly increase the RAS/RT, RWC, and soil aggregation stability of grapevines under drought stress, but they also led to the development of an extensive root system and greater total dry weight. Thus, we may conclude that the main role of EPS production under drought conditions is to augment the levels of rhizosphere soil nutrients and regulate water.

Drought stress can induce the excessive production of reactive oxygen species (ROS), which could impair or degrade normal cell metabolism functioning *via* oxidative damage of DNA, membrane proteins, and lipids, ultimately limiting the growth of plants (Song et al., 2014; Yang et al., 2015). Notably, MDA, the final decomposition product of membrane lipid peroxidation, could be used to gauge the degree to which a plant has been damaged in the face of adversity (Peng et al., 2020). This view is supported by our study’s results, in that imposing drought significantly increased the MDA in grapevine. To overcome the negative effects caused by ROS, the antioxidant defense systems of plants, which include SOD, POD, and CAT enzymes, are activated under drought stress (Jesus et al., 2015) to eliminate excessive ROS. In our study, the SOD, POD, and CAT activities of the grapevines inoculated under drought stress were significantly higher than those without inoculation and accompanied by a decreased MDA. Therefore, our results support the view that the PGPR inoculations are very effective at reducing oxidative damage under different stress conditions (Barnawal et al., 2016a; Zhang et al., 2020). While facing drought stress conditions, this study’s experimental injections of *P. corrugata*, *E. soli*, and their mixed inoculation into the rhizosphere were able to significantly reduce the MDA content of the grapevines. Accordingly, we anticipate the three inoculums could reduce the MDA content by increasing the activity of SOD and POD under drought conditions, thereby improving the antioxidant defense activity and drought resistance of grapevines in field settings.

Further, the hormonal balance of a plant can be modulated by phytohormones produced by PGPRs. This could regulate morphological and physiological characteristics of plants indirectly, thus promoting plant growth under stressful conditions. Our results revealed that drought stress significantly decreased IAA, whereas the two strains have stronger capacity to promote the synthesis of IAA (*P. corrugata*: 10.29μg/ml; *E. soli*: 11.14μg/ml). Inoculation with PGPRs increased the endogenous IAA production compared with the control under both normal and drought conditions. In this way, the root biomass and surface area would be increased, which then promoted the uptake of water and nutrients, thus ensuring plant growth and survival when incurring drought stress. This is supported by other findings that IAA is one of the vital factors which can alleviate exogenous stress upon plants, by stimulating plant development, significantly increasing dry matter content, shoot or root lengths, and affecting the absorption of nutrients by plants (Baris et al., 2014; Pal et al., 2019; Zerrouk et al., 2019). Previous studies have also shown that IAA could promote the growth of cotton (Kapgate et al., 1989), soybeans (Sarkar et al., 2002), and *Brassica juncea* (Mir et al., 2020). In addition, some studies demonstrated IAA does not only regulate growth of the root system to increase the absorption of water and nutrients, but also enhance stomatal conductance, photosynthetic rate, and the activity of antioxidant enzymes by removing excess ROS (Shi et al., 2014; Li et al., 2019).

Another important stress hormone, ABA, has been well-studied for how it enhances drought tolerance in plant through regulating stress-induced genes as well as signaling the stomatal closure under drought conditions (During, 1984; Jochum et al., 2019). Diminished stomatal conductance is beneficial for lessening transpiration, thus reducing water losses, but it may lead to insufficient CO_2_ and a lowering of the plant photosynthesis rate. Our results showed that drought significantly increased ABA and indirectly decreased Gs to reduce transpiration in grapevines, thereby increasing their WUE. Inoculation with PGPRs significantly decreased the ABA concentration in grapevines, compared with non-inoculated grapevines under drought stress. Similar results were reported for maize (Porcel et al., 2014) and tomato (Belimov et al., 2015). Therefore, we speculated that PGPRs with ACC deaminase activity might alleviate the diminished photosynthetic characteristics due to drought stress by reducing the content of ABA. Our experiment confirmed this hypothesis, in that the chlorophyll content of leaves, Pn, Gs, and WUE were all significantly increased by inoculation strains under drought conditions. Those results are supported by Liu et al. (2019), who determined the photosynthetic characteristics of *Sambucus williamsii* Hance seedling leaves in response to inoculation with *Acinetobacter calcoaceticus* X128. Therefore, PGPRs can promote plant growth and increase dry matter accumulation by increasing the photosynthetic rate (Liu et al., 2013).

## Conclusion

This study shows that several ACC deaminase-producing rhizobacteria exhibit high tolerance to drought stress. Hence, roots of wine grapevines can be selected as a resource to isolate PGPRs that might be used to protect plants from drought stress impacts. Our study suggests that inoculations with *Pseudomonas corrugate* DR3 and *E. soli* DR6 isolated from rhizosphere soil of wine grape could effectively alleviate drought stress damage and promote the growth of 1-year-old own-rooted grapevines, with their mixed inoculation showing the best promotion effects. The mechanisms by which PGPRs alleviate environmental stress and promote plant growth rely on complex combination of numerous pathways. These two strains improved soil nutrients and then promoted plant growth by contributing to enhanced nitrogen fixation and phosphate solubilization. Additionally, the PGPRs’ ability to partly regulate phytohormones and induce the ROS defense system indirectly affects biochemical and physiological properties of grapevines, ameliorating the drought stress incurred by plants. Therefore, *P. corrugata* DR3 and *E. soli* DR6 both offer great potential as biological fertilizers in arid regions for the sustainable development of the grape industry.

## Data Availability Statement

The datasets presented in this study can be found in online repositories. The names of the repository/repositories and accession numbers can be found at: https://www.ncbi.nlm.nih.gov/, MK774791; MK346043; MK411217; MK611658; MK611660; MK611659; and MK611665.

## Author Contributions

XL designed the experiments and was responsible for the manuscript revision. GC performed the experiment and laboratory analysis. BD and LL co-wrote the paper. CS-Z, YL, HM, and WL analyzed the data and edited the manuscript. All authors contributed to the article and approved the submitted version.

## Conflict of Interest

The authors declare that the research was conducted in the absence of any commercial or financial relationships that could be construed as a potential conflict of interest.

## Publisher’s Note

All claims expressed in this article are solely those of the authors and do not necessarily represent those of their affiliated organizations, or those of the publisher, the editors and the reviewers. Any product that may be evaluated in this article, or claim that may be made by its manufacturer, is not guaranteed or endorsed by the publisher.

## Footnotes

^1^ http://www.ncbi.nlm.nih.gov/
